# Corticosteroid Treatment Influences TA-Proteinuria and Renal Survival in IgA Nephropathy

**DOI:** 10.1371/journal.pone.0158584

**Published:** 2016-07-14

**Authors:** Cristina Sarcina, Carmine Tinelli, Francesca Ferrario, Bianca Visciano, Antonello Pani, Annalisa De Silvestri, Ilaria De Simone, Lucia Del Vecchio, Veronica Terraneo, Silvia Furiani, Gaia Santagostino, Enzo Corghi, Claudio Pozzi

**Affiliations:** 1 Department of Nephrology and Dialysis, E. Bassini Hospital, Cinisello Balsamo, Milan, Italy; 2 Biometry and Clinical Epidemiology Unit, Policlinico San Matteo, Pavia, Italy; 3 Department of Nephrology and Dialysis, G. Brotzu Hospital, Cagliari, Italy; 4 Department of Nephrology, Dialysis and Renal Transplant A. Manzoni Hospital, Lecco, Italy; Emory University, UNITED STATES

## Abstract

The clinical course of IgA nephropathy (IgAN) and its outcome are extremely variable. Proteinuria at baseline has been considered one of the most important risk factors. More recently, mean proteinuria of follow-up (time-average proteinuria: TAp) was described as a stronger marker of renal survival, suggesting to consider it as a marker of disease activity and response to treatment. We evaluated predictors of renal survival in IgAN patients with different degrees of renal dysfunction and histological lesions, focusing on the role of the therapy in influencing TAp. We performed a retrospective analysis of three prospective, randomized, clinical trials enrolling 325 IgAN patients from 1989 to 2005. Patients were divided into 5 categories according to TAp. The primary endpoint of the 100% increase of serum creatinine occurred in 54 patients (16.6%) and renal survival was much better in groups having lower TAp. The median follow up was 66.6 months (range 12 to 144). The primary endpoint of the 100% increase of serum creatinine occurred in 54 patients (16,6%) and renal survival was much better in groups having lower TA proteinuria. At univariate analysis plasma creatinine and 24h proteinuria, systolic (SBP) and diastolic (DBP) blood pressure during follow-up and treatment with either steroid (CS) or steroid plus azathioprine (CS+A) were the main factors associated with lower TAp and renal survival. At multivariate analysis, female gender, treatment with S or S+A, lower baseline proteinuria and SBP during follow-up remained as the only variables independently influencing TAp. In conclusion, TA-proteinuria is confirmed as one of the best outcome indicators, also in patients with a severe renal insufficiency. A 6-month course of corticosteroids seems the most effective therapy to reduce TAp.

## Introduction

IgA nephropathy (IgAN) is the most common glomerulonephritis in the world, particularly in males [1]. The clinical course of the disease and its outcome are extremely variable, with some patients (pts) having stable renal function for decades and others rapidly progressing to end-stage renal disease (ESRD). Proteinuria at the time of renal biopsy has always been considered one of the most important prognostic factors [2–6], but recently Reich et al found that pts who achieved and sustained proteinuria levels below 1 g/day during follow up had a favourable outcome, regardless of baseline proteinuria levels, suggesting the importance of time-average proteinuria (TAp) as a marker of disease activity and response to treatment [7].

In 2004 we reported the long-term results of a randomized, controlled trial of 86 IgAN pts who received corticosteroids or supportive therapy. Accordingly, the changes in proteinuria during follow-up predicted the outcome better than absolute proteinuria values at onset [8]. Similar findings were found in a recent, large trial of 207 pts, who were randomized to steroids plus azathioprine or steroids alone and followed up for a median of 5 years [9]. Interestingly, this observation was true for pts who either received immunosuppressant treatment or not. Recently, Coppo et al. have confirmed the value of TAp in 1147 patients of the VALIGA study [10].

Histological features may also be helpful in identifying pts who are at risk of progression. However, no data are available to unquestionably clarify the role of different histological lesions, either using the World Health Organization (WHO) criteria modified by Churg and Sobin [11] or the recent Oxford classification [12].

The aim of the present study is to evaluate predictors of renal survival in IgAN pts with different degrees of renal dysfunction, particularly focusing on the role of the therapy and its influence on TAp.

## Patients and Methods

Our study is a retrospective analysis of three different clinical trials; no ethics statement was required.

This clinical investigation has been conducted according to the principles expressed in the Declaration of Helsinki; informed consent have been obtained from the participants.

The study was approved by the ethics committees of all hospitals in which patients were enrolled for the first three studies of treatment. All patient records were anonymized and de-identified prior to analysis.

We performed a retrospective analysis of three prospective, randomized, clinical trials enrolling 325 IgAN pts from 1989 to 2005 in 27 centres (26 in Italy and one in Switzerland) [9,13,14]. These studies were designed and coordinated by our Center, the schemes of steroid administration was similar and we were able to access all the data included in the database. Therefore we report results of a median follow-up of 66.6 months (range 12 to 144). The inclusion and exclusion criteria of the three studies and treatment schedules have already been described elsewhere and are reported in Table 1 [8,9,13,14]. Briefly, the first trial enrolled 86 pts having proteinuria of 1–3.5 g/day and serum creatinine ≤1.5 mg//dl; the second one enrolled 207 pts with proteinuria ≥1 g/day and serum creatinine ≤ 2 mg/ dl (10 pts were excluded from this evaluation, because they dropped out in the first 12 months); the third one enrolled 42 pts with proteinuria ≥1 g/day and serum creatinine > 2 mg/ dl (4 pts were excluded because they dropped out in the first 12 months). Pts were evaluated at baseline and at the end of the 6-month treatment; afterwards, controls were made six months later and then every year. For every single patient TAp was calculated and pts were divided into 5 categories according to TAp values, as described by Reich^7^: <0.3g/day (group 1), 0.3–0.9 g/day (group 2), 1–1.9 g/day (group 3), 2–2.9 g/day (group 4), ≥ 3 g/day (group 5). We defined as renal insufficiency an estimated glomerular filtration rate (eGFR) <60 ml/min/1.73 m^2^ of body-surface area, calculated with the use of the four-variable Modification of Diet in Renal Disease formula.

**Table 1:** Characteristics of the three considered trials.

Trial	Inclusion criteria	Treatment	Endpoints	Renal survival	Median Proteinuria
1.Corticosteroids in IgA nephropathy: a randomised controlled trial^13^	Histological diagnosis of IgA nephropathy, age 15–69 years, proteinuria 1–3.5 g/d, plasma creatinine ≤ 1.5 mg/dl	A. Supportive treatment (43 pts)	50% increase from baseline plasma creatinine	After 5 years: 50% increase pl.cr: 21% steroid vs 33% supportive t. groups	Steroid treatment: baseline 2 g/d, after 6 months 1 g/d, after 12 months 0.6 g/d
		B. 1 g methylprednisolone i.v. for 3 days at the beginning of months 1,3,5, plus oral prednisone 0.5 mg/kg on alternate days for 6 months (43 pts)	100% increase from baseline plasma creatinine	100% increase pl.cr: 2% steroid vs 21% supportive t. groups	Supportive treatment: baseline 1.8 g/d, after 6 months 1.5 g/d, after 12 months 1.2 g/day
			evolution of proteinuria over time		
(Corticosteroid effectiveness in IgA nephropathy: long-term results of a randomized, controlled, trial)^8^		A.43 pts		After 10 years: 100% increase pl.cr: 2.3% steroid vs 30.2% supportive t. groups	Median proteinuria during 10 y FU: 0.8 g/day in steroid vs 1.7 g/d in supportive t. groups
		B. 43 pts			
2.Addition of Azathioprine to Corticosteroids does not benefit patients with IgA nephropathy^9^	Histological diagnosis of IgA nephropathy, adult patients, proteinuria ≥ 1 g/d, plasma creatinine ≤ 2.0 mg/dl	A. 1 g methylprednisolone i.v. for 3 days at the beginning of months 1,3,5, plus oral prednisone 0.5 mg/kg on alternate days for 6 months plus 1.5 mg(kg per day of azathioprine (101 pts)	50% increase from baseline plasma creatinine	After 5 years: 50% increase pl.cr: 88% S+AZT vs 89% S group	after 5 y FU: S+AZT: from 2.10 to 1.16 g/d
		B. The same steroid schedule alone (106 pts)	evolution of proteinuria over time	After 7 years 50% increase pl.cr: 83% S+AZT vs 84% S groups	S: from 1.95 to 0.98 g/d
3.IgA nephropathy with severe chronic renal failure: a randomized controlled trial of corticosteroids and azathioprine^14^	Histological diagnosis of IgA nephropathy, adult patients, proteinuria ≥ 1 g/d, plasma creatinine > 2.0 mg/dl	A. 1 g methylprednisolone i.v. for 3 days at the beginning of months 1,3,5, plus oral prednisone 0.5 mg/kg on alternate days for 6 months plus 1.5 mg(kg per day of azathioprine, followed by oral prednisone 0.2 mg/kg every other day for 6 months (20 pts)	50% increase from baseline plasma creatinine	After 5 years: 50% increase pl.cr: S+AZT 58% vs 63% S group	Median proteinuria after 5 years of FU: S+AZT: from 3.2 to 2.73 g/d
		B. The same steroid schedule alone (26 pts)	evolution of proteinuria over time		S: from 2.0 to 1.05 g/d

### Evaluation of outcomes

For the present analysis, we considered the time to the doubling of serum creatinine from baseline as the primary endpoint; secondary endpoint was the time to ESRD.

### Factors influencing Tap

We investigated possible risk factors that could influence TAp: age, gender, blood pressure at baseline and during follow-up, histological lesions, RAS blockers and treatment with steroids or steroids plus azathioprine (treatment schedules have been described elsewhere [9,13,14]). Systolic (SBP) and diastolic blood pressure (DBP) values were recorded at the same time of proteinuria and for each patient mean values were reported. Histological lesions were evaluated in 273 pts, in whom renal biopsy was performed during three months preceding enrolment. In the remaining 52 pts, renal biopsy was performed more than 6 months before and the histological evaluation was not considered useful for the present analysis. At the time of enrolment, histological specimens were evaluated by the Centres using the WHO criteria as modified by Churg and Sobin (grade I: minimal glomerular lesions; grade II: active glomerular, tubular, and interstitial lesions; grade III: active and chronic lesions) [11]. For technical reasons, we were not able to perform the Oxford classification (a significant number of pts underwent renal biopsy more than ten years before and despite adequate storage, images often fade and become unreadable after 10–15 years).

### Statistical analysis

The Shapiro-Wilk test was used to test the normal distribution of quantitative variables. When quantitative variables were normally distributed, the results were expressed as mean values and standard deviation (SD), otherwise median and interquartile range (IQR) plus 25^th^-75^th^ percentiles were reported; qualitative variables were summarized as counts and percentages. To avoid bias due to missing follow-up measures, a multiple imputation model for missing data was fitted taking into account sex, age, serum creatinine and proteinuria at baseline and histological grading. The descriptive analysis of the time of reaching the primary and secondary endpoint were performed by computing survival curves according to the Kaplan-Meier method; survival rates together with 95% confidence intervals (CI) were. For highly skewed variables, log transformation was applied. Univariate and multivariate Cox proportional hazards models were used to determine the factors associated with the risk of reaching the doubling of serum creatinine from baseline (multifailure model) or ESRD. Results were expressed as hazard-rate (HRs) with CI. The multivariate models were built considering that the main interest was the identification of prognostic factors, in addition to TA-proteinuria, present at the enrollment and statistically significant at univariate analysis. Also interaction between treatment and histological grade or basal proteinuria was taken into account. Proportional hazard assumption was verified on the basis of Schoenfeld residuals, testing the null hypothesis of zero slope in a generalized linear regression of the scaled Schoenfeld residuals on time which is equivalent to testing that the log hazard-ratio function is constant over time [15].

The associations of TA-proteinuria with sex, age, histological grading, treatment, 24h-proteinuria and serum creatinine at baseline and BP values were performed with regression univariate and multivariate models for repeated measures. In the final model, only those variables statistically significant at the 0.05 level at univariate analysis were retained. The results are expressed as mean change (coefficients with their 95% CI) per unit of the independent variable. P values <0.05 were considered to be statistically significant. Data analysis was performed with STATA statistical package (release 12, 2011, Stata Corporation, College Station, Texas, USA).

## Results

### Patients

The main characteristics of the 325 pts at baseline are reported in Table 2. The majority were male (74.5%) and Caucasians (97.9%). Mean age was 38.5±12.9 years (range 18 to 70), mean proteinuria was 2.35±1.5 g/day and mean serum creatinine was 1.45±0.7 mg/dl. Renal insufficiency (eGFR <60 ml/min/1.73 m^2^) was found in 42.4% of the pts. More than half of subjects had severe histological lesions (grade III). 164 pts (50.5%) were receiving a RAS blocker at baseline. According to the original study protocols, 171 pts were assigned to receive corticosteroids (CS), 111 to corticosteroids plus azathioprine (CS+A) and 43 to only supportive treatment (NT). In the three treatment groups, no differences were found about age, sex, histological grading and systolic and diastolic BP values at baseline. Conversely, serum creatinine, 24h-proteinuria and the use of RAS blockers were lower in the NT than in the CS and CS+A groups, likely because NT pts were enrolled in the oldest^12^ of the three studies that had different inclusion criteria. Pts were followed up for a median of 66.6 months (IQR: 48–84; 3; range 12 to 144).

**Table 2:** Characteristics at baseline, after 6 months and during follow-up according to the treatment.

Variable	All (325 pts)	NT (43 pts)	CS (171 pts)	CS+A (111 pts)	P value#
**Baseline**					
Age	38.5 (12.9)	38.9 (12.9)	39.4 (13.4)	36.9 (12.1)	0.2802
Male sex	242 (74.5)	31 (72.1)	124 (72.5)	87 (78.4)	0.506
Histological Grade I	24 (8.7)	2 (4.6)	14 (9.7)	8 (9.1)	
Histological Grade II	105 (38.2)	21 (48.8)	50 (34.7)	34 (38.6)	
Histological Grade III	146 (53.1)	20 (46.5)	80 (55.6)	46 (52.3)	0.516*
RAS blockers	164 (50.5)	5 (11.6)	92 (53.8)	67 (60.4)	< 0.001^1^
Systolic BP mmhg	132.9 (16.6)	133.4 (20.7)	133.2 (16.0)	132.2 (16.0)	0.634
Diastolic BP mmhg	82.7 (10.4)	83.4 (12.3)	82.8 (10.1)	82.2 (10.1)	0.787
Serum creatinine mg/dl	1.45 (0.7)	1.06 (0.2)	1.52 (07)	1.50 (0.6)	<0.001^2^
eGFR > 60 ml/min/1.73m^2^	187 (57.5)	37 (86)	90 (52.6)	60 (54)	
eGFR 30–60 ml/min/1.73m^2^	109 (33.5)	6 (14)	63 (36.8)	40 (36)	
eGFR < 30 ml/min/1.73m^2^	29 (8.9)	0	18 (10.5)	11 (10)	0.002^11^
Proteinuria g/day	2.35 (1.5)	1.88 (0.7)	2.20 (1.0)	2.76 (2.1)	<0.001^3^
Renal insufficiency (eGFR < 60 ml/min)	138 (42.4)	6 (14.0)	81 (47.3)	51 (46.0)	0.016^4^
**After 6 months**					
Proteinuria g/day—Median (IQR)	0.9 (0.5–1.6)	1.7 (1.1–2.4)	0.8 (0.4–1.2)	1.0 (0.5–1.7)	<0.001^5^
≤0.3 (G1)	36 (11.6)	0 (0)	21 (12.9)	15 (14.4)	
>0.3 to <1.0 (G2)	125 (40.3)	9 (20.9)	78 (47.8)	38 (36.5)	
1.0 to < 2.0 (G3)	94 (30.3)	16 (37.2)	47 (28.8)	31 (29.8)	
2.0 to < 3.0 (G4)	32 (10.3)	10 (23.3)	12 (7.4)	10 (9.6)	
≥ 3.0 (G5)	23 (7.4)	8 (18.6)	5 (3.1)	10 (9.6)	<0.001*^6^
Serum creatinine mg/dl	1.4 (0.7)	1.11 (0.3)	1.45 (0.7)	1.45 (0.7)	<0.001^7^
**Follow-up**					
TAp g/day—Median (IQR)	0.7 (0.4–1.3)	1.4 (0.8–2.4)	0.6 (0.3–1.1)	0.6 (0.3–1.2)	<0.001^8^
≤0.3 (G1)	58 (17.8)	1 (2.3)	35 (20.5)	22 (19.8)	
>0.3 to <1.0 (G2)	144 (44.3)	12 (27.9)	80 (46.8)	52 (46.8)	
1.0 to < 2.0 (G3)	77 (23.7)	14 (32.6)	44 (25.7)	19 (17.1)	
2.0 to < 3.0 (G4)	22 (6.8)	7 (16.3)	9 (5.3)	6 (5.4)	
≥ 3.0 (G5)	24 (7.4)	9 (20.9)	3 (1.7)	12 (10.8)	<0.001*^9^
SBP mmhg	131.9 (17.2)	136.9 (23.5)	131.1 (14.4)	131.1 (18.2)	0.1252
DBP mmhg	82.1 (10.5)	84.3 (12.7)	81.6 (9.0)	81.9 (11.6)	0.311
Serum creatinine mg/dl	1.5 (0.75)	1.2 (0.5)	1.5 (0.8)	1.5 (0.7)	0.036
RAS blockers	259 (79.7)	18 (41.9)	140 (81.9)	101 (91.0)	<0.001^10^

Data are n (%) for qualitative variable and Mean (SD), unless otherwise noted, for quantitative variables. NT: not treatment; CS: Steroids; A: azathioprine; # P value refers at differences among three treatment group;

* P value for differences also among all proteinuria groups. IQR: interquartile range.

^1^ NT vs CS and NT vs CS+A: p<0.001, CS vs CS+A: p = 0.278;

^2^ NT vs CS and NT vs CS+A: p<0.001, CS vs CS+A: p = 0.985;

^3^ for all comparisons: p <0.001;

^4^ NT vs CS: p = 0.005, NT vs CS+A: p0.043, CS vs CS+A: p = 0.334;

^5^ for all comparisons: p <0.001;

^6^ NT vs CS and NT vs CS+A: p<0.001, CS vs CS+A: p = 0.127;

^7^ NT vs CS and NT vs CS+A: p<0.001, CS vs CS+A: p = 0.973;

^8^ NT vs CS and NT vs CS+A: p<0.001, CS vs CS+A: p = 0.002;

^9^ NT vs CS and NT vs CS+A: p<0.001, CS vs CS+A: p = 0.014;

^10^ NT vs CS and NT vs CS+A: p<0.001, CS vs CS+A: p = 0.034;

^11^ CS VS CS+A:P = 0,002.

### Time-average proteinuria and renal survival

According to TAp, pts were divided into five groups (G): G1 (TAp <0.3 g/24h) 58 pts (17.8%), G2 (TAp 0.3–0.9 g/24h) 144 pts (44.3%), G3 (TAp 1–1.9 g/24h) 77 pts (23.7%), G4 (TAp 2–2.9 g/24h) 22 pts (6.8%), G5 (Tap ≥ 3 g/24h) 24 pts (7.4%). The numbers of patients at risk during follow-up were as follows: at 6 months, 325 patients; at 1 year, 325 patients; at 2 years, 309 patients; at 3 years, 285 patients; at 4 years, 249 patients; at 5 years, 208 patients; at 6 years, 171 patients; at 7 years, 119 patients; at 8 years, 70 patients; at 9 years, 43 patients; and at 10 years, 27 patients.

**Fig 1:**
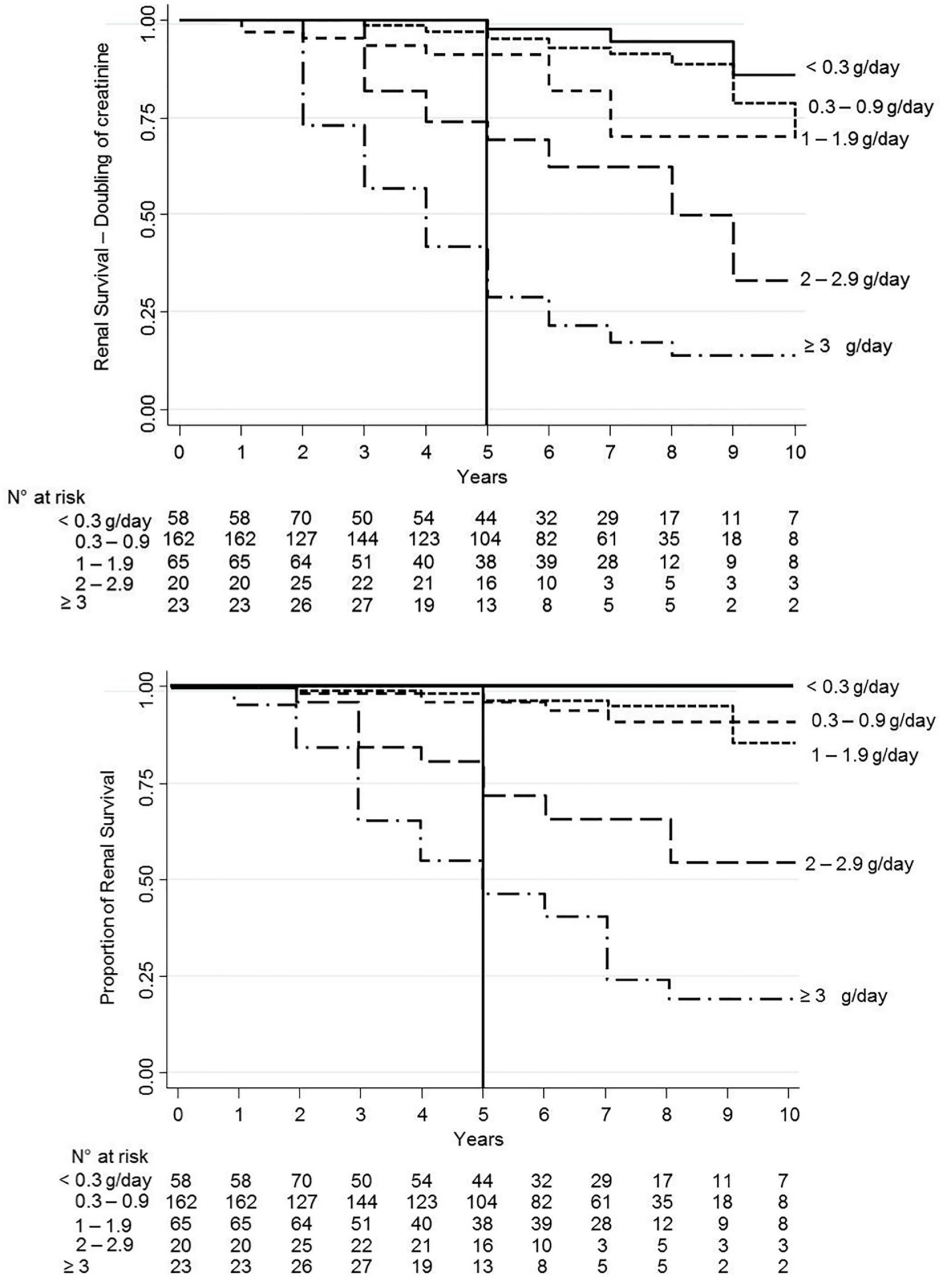
Renal survival. (A) Kaplan-Meier estimates of proportion of renal survival (absence of creatinine doubling) in the 325 patients by TAp groups. Estimated 5-year overall survival rates were 97.7% (95% CI 84.9–99.7) for patients with <0.3 g/day; 95.1% (95% CI 89.4–97.8) for patients with 0.3–0.9 g/day, 92.1% (95% CI 79.8–96.3) for those with 1–1.9 g/day, 69.4% (95% CI 46.3–84.1) for those with 2–2.9 g/day and 29.0% (95% CI 14.6–45.1) for those with ≥3 g/day. (B) Kaplan-Meier estimates of proportion of renal survival (absence of ESRD) in the 325 patients by TAp groups. Estimated 5-year overall survival rates were 100% (95% CI: not calculable) for patients with <0.3 g/day; 96.5% (95% CI 90.1–98.7) for patients with <0.3–0.9 g/day, 95.6% (95% CI 84.3–99.2) for those with 1–1.9 g/day, 69.1% (95% CI 45.6–84.0) for those with 2–2.9 g/day and 46.9% (95% CI 27.7–63.9–45.1) for those with ≥3 g/day.

The primary endpoint of the 100% increase of serum creatinine from baseline occurred in 54 pts (16.6%) (Table 3), with a five-year renal survival of 85.9% (95% IC: 81.3–89.4). Renal survival for this endpoint decreased proportionally with the increase of TAp: TAp1 97.7% (95% CI: 84.9–99.7), TAp2 95.1% (95% CI: 89.4–97.8), TAP3 92.1% (95% CI: 79.8–96.3), TAp4 69.4% (95% CI: 46.3–84.1), TAp5 29% (95% CI: 14.6–45.1) (Fig 1A).

**Table 3:** 100% increase of creatinine (54 patients—66 events): risk factors at univariate analysis.

	Univariate		
	HR	95% CI	p
Sex (F vs M)	0.67	0.374–1.22	0.19
Age	1.008	0.991–1.026	0.37
Histological grade			
I	1		
I+II	2.03	0.35–11.84	0.43
III	4.15	0.73–23.46	0.11
Treatment			
No treatment	1		
CS	0.50	0.26–0.99	0.04
CS+A	0.54	.03–1.09	0.08
RAS blocker at baseline	2.00	1.2–3.33	<0.01
RAS blocker during FU	1.61	.76–3.38	0.2
Lipid lowering therapy	1.48	.83–2.67	0.18
Proteinuria baseline (g/24h)	1.39	1.24–1.57	4.08e-08
1–1.99 gr/24h	1		
2–2.99 gr/24h	1.51	.8–2.83	0.19
≥ 3 gr/24h	3.35	1.87–5.97	<0.0001
eGFR (per ml/min/1.73m2) at baseline	0.98	0.97–0.99	<0.001
> 60 ml/min/1.73m2	1		
30–60 ml/min/1.73m2	2.57	1.46–4.53	0.001
< 30 ml/min/1.73m2	6.86	3.53–13.3	<0.001
SBP baseline	0.99	.98–1.0	0.72
DBP baseline	0.99	.98–1.02	0.96
Proteinuria after 6 months g/24h	1.66	1.45–1.89	2.10e-13
< 0.3	1.75	.39–7.81	0.46
0.3–0.99	3.89	.91–16.5	0.06
1–1.99	10.45	2.41–45.2	0.001
2–2.99	14.14	3.04–65.8	<0.001
≥ 3			
SBP after 6 months	1.00	.98–1.02	0.90
DBP after 6 months	1.03	1.00–1.05	0.01
Mean proteinuria (FU) g/24h	1.84	1.65–2.07	<.0001
Varying roteinuria (FU) g/24h	1.48	1.38–1.58	<0.001
SBP (during follow up)	1.03	1.01–1.04	<0.0001
DBP (during follow up)	1.06	1.04–1.08	3.07e-09

The secondary endpoint (ESRD) occurred in 39 pts (12%) (Table 4). Renal survival was of 88.9% at five years (Fig 1B); it reduced in according to increasing of TA proteinuria. Also for this endpoint renal survival decreased proportionally with the increase of Tap, although without differences between TAp2 and TAp3 in the first 5 years: TAp1 100%, TAp2 96.5% (95% CI: 90.1–98.7), TAp3 95.6% (95% CI: 84.3–99.2), TAp4 69.2% (95% CI: 45.6–84.0), TAp5 46.9% (95% CI: 27.746.9). The prognostic value of Tap was also confirmed in pts with an eGFR lower than 30 ml/min, even if in this small group of patients a TAp > 2 g/d was more frequently observed (Table 5).

**Table 4:** End-stage (39 patients): risk factors at univariate analysis (Cox model with multiple imputation).

	Univariate		
	HR	95% CI	p
Sex (F vs M)	.37	.15-.96	.04
Age	1.01	.99–1.04	.20
Histological grade			
I	Np		
I+II	180961.4	0–1.38e+15	.37
III	556500.7	0–3.88e+15	.25
Treatment			
No treatment	1		
CS	.57	.22–1.45	.23
CS+A	.85	.33–2.17	.73
RAS blockers at baseline	2.3	1.16–4.53	0.017
RAS bl. baseline +/- during FU	1.41	.62–3.21	.41
Lipid lowering therapy	1.17	.54–2.52	.69
Proteinuria baseline (g/24h)	1.52	1.34–1.73	<0.0001
1–1.99 g/24h	1		
2–2.99 g/24h	1.59	.63–4.02	.32
≥ 3 g/24h	5.86	2.68–12.7	<0.0001
SBP _baseline (mmHg)	1.02	.99–1.03	.08
DBP_baseline (mmHg)	1.00	.97–1.03	.80
eGFR (per ml/min/1.73m^2^) at baseline	0.94	0.93–0.96	<0.001
> 60 ml/min/1.73m^2^	1		
30–60 ml/min/1.73m^2^	5.67	2.08–15.5	0.001
< 30 ml/min/1.73m^2^	28.3	10.5–76.3	<0.001
Proteinuria at 6 months (g/24 h)	1.83	1.59–2.10	<0.0001
< 0.3	np		
0.3–0.99	1		
1–1.99	.955	.35–2.57	0.92
2–2.99	5.92	2.49–14.07	<0.0001
≥ 3	11.11	4.45–27.68	<0.0001
Serum Creatinine at 6 months (per mg)	4.41	3.22–6.03	<0.0001
SBP at 6 months (mmHg)	1.01	.99–1.03	.23
DBP at 6 months (mmHg)	1.03	1.00–1.06	.03
Mean proteinuria during FU (g/24h)	1.91	1.67–2.19	<0.0001
Varying proteinuria during FU (gr/24h)	1.47	1.35–1.59	<0.0001
< 0.3	Np		
0.3–0.99	1		
1–1.99	1.86	.54–6.45	0.32
2–2.99	10.84	3.62–32.42	<0.0001
≥ 3	24.47	9.14–65.47	<0.0001
Serum Creatinine (during FU) (mg/dl)	1.038	1.02–1.05	<0.0001
SBP (during follow_up) (mmHg)	1.033	1.02–1.05	.00006
DBP (during follow_up) (mmHg)	1.07	1.04–1.09	<0.0001

**Table 5:** Change in eGFR during FU according to TAp in all patients and in patients with eGFR <30 ml/min.

Time-average Proteinuria (g/d)	Patients N° (%)	Mean GFR at baseline (ml/min)	Mean GFR at last visit (ml/min)	Variation of GFR (ml/min)	Mean FU (years)
**All patients (study 1, 2, 3)**					
**< 0.3**	22 (7%)	78.8	79.1	+ 0.3	5.8
**0.3–0.9**	162 (51.4%)	73.9	70.6	- 3.3	6.3
**1.0–1.9**	86 (27.3%)	75.6	61.7	- 13.9	5.5
**2.0–2.9**	21 (6.7%)	66.2	40.0	- 26.2	5.6
**≥ 3**	24 (7.6%)	64.4	21.5	- 42.9	3.9
**Patients of study 3**					
**< 0.3**	2 (4.9%)	21.5	29.0	+7.5	4.5
**0.3–0.9**	17 (41.5%)	28.3	27.5	-0.8	5.4
**1.0–1.9**	11 (26.8%)	28.9	12.9	-16	5.0
**2.0–2.9**	6 (14.6%)	20.3	7.5	-12.8	3.5
**≥ 3**	5 (12.2%)	29.2	6.3	-22.9	3.4

### Factors associated to renal survival

#### Univariate analysis

*Primary Outcome–*Table 3 describes univariate analysis for the primary endpoint. In particular, we found that the higher risk of reaching the doubling of serum creatinine was present in pts having higher 24h proteinuria, either at baseline (for each more gram: HR 1.4, 95% CI: 1.2–1.6, p<0.001) or after 6 months (for each more gram: HR: 1.65, 95% CI: 1.4–1.9, p<0.0001), or during the whole follow up (for each more gram: HR 1.8, 95% CI: 1.6–2.1, p<0.0001). Other significant factors influencing the risk of reaching the primary end-point were the degree of renal dysfunction at baseline (HR 2.57, 95% CI 1.5–4.5 p = 0.001 for eGFR at baseline 30–60 ml/min/1.73m^2^; HR 6.86, 95% CI 3.5–13.3, p<0.001 for eGFR at baseline <30 ml/min/1.73m^2^), the use of RAS blocker at baseline and DBP and SBP during follow-up. Also use of CS resulted significant (p = 0.04). Sex, age, SBP and DBP at baseline and histological grading were not found statistically significant.

*Secondary Outcome—*Similar findings were obtained for the risk of ESRD: 24h proteinuria at baseline ≥ 3 g (HR 5.85, 95% CI: 2.7–12.7, p<0.0001), at 6 months and during follow-up, eGFR < 60 ml/min/1.73m^2^ at baseline (HR 5.67, 95% CI 2.1–15.5, p<0.001 for eGFR at baseline 30–60 ml/min/1.73m^2^; HR 28.3, 95% CI 10.5–76.3, p<0.001) use of RAS blockers at baseline, sex, BP values measured during follow-up were all significantly associated to risk of ESRD (Table 4).

#### Multivariate analysis

*Primary outcome—*The findings of multivariate Cox regression analysis were: varying proteinuria during FU (HR 1.49, CI 95% 1.4–1.6, p<0.001), proteinuria ≥ 3 g/day at baseline (HR 2.53, 95% CI 1.34–4.79, p < 0.0001), eGFR < 60 ml/min/1.73m^2^ (HR 3.23, 95% CI 1.6–6.7, p = 0.002 for eGFR 60–30 ml/min/1.73m^2^; HR 7.28, 95% CI 3.1–9.5, p<0.001 for eGFR < 30 ml/min/1.73m^2^), treatment with CS (HR 0.36, 95% CI 0.1–0.9, p = 0.031) and combined treatment with CS+A (HR 0.30, 95% CI 0.1–0.7, p = 0.01) were independent factors related to progression to the doubling of serum creatinine from baseline.

*Secondary Outcome—*The findings of multivariate Cox regression analysis were: varying proteinuria during FU (HR 1.58, CI 95% 1.4–1.8, p<0.001), eGFR < 60 ml/min/1.73m^2^ (HR 8.3, 95% CI 4.9–14.1, p<0.001 for eGFR 60–30 ml/min/1.73m^2^; HR 30.72, 95% CI 17.4–35.0, p<0.001 for eGFR < 30 ml/min/1.73m^2^), treatment with CS (HR 0.18, 95% CI 0.1–0.7, p = 0.01) and combined treatment with CS+A (HR 0.13, 95% CI 003–0.5, p = 0.001).

### Factors associated to TAp

Since renal survival inversely correlated with TA-proteinuria, we searched for factors possibly influencing TAp (Table 6). At univariate analysis, histological lesions of grade III, baseline 24 h proteinuria, serum creatinine at baseline and after 6 months, SBP and DBP at baseline and during follow-up negatively influenced TAp. Conversely, female gender, treatment with either CS (average decrease of 0.87 g/day) or CS+A (average decrease of 0.59 g/day) were the main factors associated with lower TAp. At multivariate analysis, female gender (HR -0.2, 95% CI -0.36–0.4, p = 0,01), treatment with CS (average decrease of -1.0 g/day, 95% CI -1.4–0.61, p<0.001) or CS+A (average decrease of -0.87 g/day, 95% CI -1.3–0.48, p < 0.001), lower baseline proteinuria and SBP during follow-up remained as the only variables independently influencing TAp.

**Table 6:** Correlation between time-average proteinuria and clinical factors.

	Univariate			Multivariate		
	Coeff.	95% CI	p	HR	95% CI	p
Sex (F vs M)	-0.26	-0.45–-0.07	0.007	-0.20	-0.36–-0.4	0.016
Age at enrolment	0.004	-.003–0.13	0.28			
Histological grade						
I	reference					
I+II	0.24	-0.08–0.57	0.14	0.07	-0.20–0.33	0.615
III	0.39	0.09–0.68	< 0.001	-0.004	-0.25–0.24	0.970
Treatment: no	reference					
CS	-0.87	-1.24–-0.49	< 0.001	-1.00	-1.4–-0.61	< 0.001
CS+A	-0.59	-1.0–-0.17	0.005	-0.87	-1.3–-0.48	< 0.001
RAS blocker use_at baseline	0.16	-0.4–0.36	0.13			
Proteinuria at baseline (g/24 h)	0.40	0.30–0.50	< 0.001		0.30–0.47	< 0.001
1–1.99	reference					
2–2.99	0.26	0.07–0.44	0.007			
≥ 3	0.97	0.64–1.30	< 0.001			
Creatinine at baseline (per mg) bbaselinerea_basale	0.28	0.10–0.47	0.003	0.09	-.05–0.22	0.233
SBP at baseline (mmHg)	0.006	0.0003–0.01	0.04	-0.004	-0.009–0.001	0.116
DBP at baseline (mmHg)	0.009	0.0001–0.02	0.04	°		
Creatinine at 6 months (per mg) months	0.42	0.21–0.62	< 0.001	§		
SBP at 6 months (mmHg)	0.006	-0.004–0.02	0.23	§		
DBP at 6 months (mmHg)	0.018	0.004–0.03	0.01	§		
Serum creatinine at FU (per mg)	0.055	-0.05–0.16	0.29			
SBP at FU (mmHg)	0.019	0.01–0.03	< 0.001	0.015	0.01–0.021	< 0.001
DBP at FU (mmHg)	0.030	0.02–0.04	< 0.001	°		

Mean time-average proteinuria of reference: 1.28 (SD 1.38) g/day

^§^ no in multivariate because yet considered in the FU

° no in multivariate because correlated with sistolic BP.

According to the behaviour of proteinuria during follow-up, 118 (36.3%) pts obtained stable remission (proteinuria always below 1 g/day). Conversely, 51 (15.7%) pts had always higher and 156 (48%) fluctuating values. Of note, of the 118 pts having remission only 11 (9.5%) did not receive CS or CS +A, whereas 67 (56.4%) received CS and 40 (34.1%) CS+A. In pts with stable decrease of proteinuria, remission was observed at the end of the 6-month therapy in 70% of cases, in 19% by 1 year and only in 14% it appeared later. After 6 months, proteinuria decreased significantly in treated pts (-1.2 g/day [SD: 1.2], p<0.001 and -1.4 g/day [SD: 1.6], p<0.001g/day in pts receiving CS and CS+A, respectively), whereas it did not change significantly in those who received supportive therapy (NT 0.09 g/day [SD: 1.2], p = 0.647). After that period proteinuria behaved similarly in the three groups (data not shown). The percentage of pts with a complete (G1) or a partial (G2) remission of proteinuria at 6 months was higher among pts treated with CS and CS+A (S 60.7%, CS+A 50.9%), than in NT pts (20.9%). During the next follow up, the distribution of remission was maintained among the three groups (CS 67.3%, CS+A 66.6%, NT 30.2%).

## Discussion

TAp is one of the strongest predictors of renal survival in IgAN [7,16]. In a disease with a slow progression, like IgAN, the role of TAp on renal survival is not much visible in the first 5 years, especially among the lower values of TAp. Anyway, our data confirm this role of TAp, showing that this prognostic value persists even for patients with severely compromised renal function. This aspect has not been documented, because in previous works pts were considered together, without distinction on the basis of the degree of renal function [7,10]. Combining the data of our three, randomised, clinical trials has been a good opportunity to investigate in-depth also the influence of steroid and immunosuppressive treatment on TAp.

The pooled analysis of these three trials seems to confirm the beneficial effect of treatment in IgAN in respect of renal survival and proteinuria reduction. Indeed, at Cox’s regression analysis, treatment with corticosteroids, either alone or in combination with azathioprine, and low TAp were the main factors positively influencing renal survival when using the doubling of serum creatinine as endpoint. The association with CS treatment and ESRD was not confirmed, but the sample and the number of pts who reached ESRD were too small to enable us to show a relationship with such a strong endpoint.

The correlation with CS and renal survival is in line with a systemic review of nine RCTs with more than 500 pts, which found that a course of CS reduced the risk for renal insufficiency more than supportive therapy or ACE inhibitors alone in IgAN pts [17]. Tesar at al, recently confirmed that CS reduce the risk of progression of renal damage in IgAN patients [18]. Our findings strongly confirm the predictive role of TAp on renal survival, with pts having highest TAp (G4 and G5) displaying the worst renal survival. Compared to the results by Reich et al. [7], our pts with the highest TAp had a worse renal survival, probably because the proportion of pts with renal insufficiency at baseline was higher in our study than in the Reich’s one (44.1% vs 27.7%, respectively). On the other side, pts with complete (G1) or partial (G2) remission of proteinuria had a renal survival free from ESRD greater than 90% at 10 years. According to our findings, treatment with either CS alone or CS+A has a significant influence on the likelihood of achieving a stable remission of proteinuria during follow-up. While only 9.5% of pts of the NT group had proteinuria remission during follow-up, in treated pts this proportion was much higher (56.4% and 34.1% in the CS group and CS+A groups, respectively). These percentages are comparable to those obtained in 109 IgAN pts, who received steroids with a same regimen; similarly to our results, proteinuria remission at two years was associated to a better renal survival [19]. Recently, to predict renal outcome, various measures of proteinuria were compared, and Time-varying proteinuria resulted the best metric to account for the prognostic effects of proteinuria over time [20]. So, we tried using the Time-varying proteinuria, obtaining results similar to those observed with the TAp (Tables 3 and 4). In 2015, the results of the STOP-IgAN study, which compared the effects on renal function of an immunosuppressive and a supportive care therapy, were published [21]. Unfortunately, no data were provided on TAp and on histological lesions. Additionally, a multivariate analysis was not made in order to know the importance of the several possible variables. In any case, even this article show that only corticosteroids, and not supportive care therapy, have reduced and kept the proteinuria below 1 g/day in 36 months of FU (Appendix 1) [21].

At multivariate analysis, we found that, in addition to treatment, systolic blood pressure at follow-up was an independent factor influencing TAp. The role of blood pressure in IgAN and in chronic nephropathies in general is widely documented. Several studies have shown that high blood pressure values during follow-up predicts a faster decline of renal function [22–23]. Recently, the Kidney Disease Improving Global Outcomes (KDIGO) guidelines [16] have suggested a blood pressure target below 130/80 mmHg in proteinuric non-diabetic adults with CKD. However, the evidence supporting this recommendation was weak.

As in other chronic nephropathies with proteinuria, RAS inhibitors are considered the drug of choice. In our pts, RAS blockers were not extensively used at baseline, since the first trial was designed in the late eighties, when a strong indication to their use as nephroprotective agents was still not there. Subsequently, following new evidences accumulating in the literature [24], the proportion of pts who received RAS blockers had increased, reaching 80% at the last observation and the use increased in the three different treatment groups in homogeneous way. Oddly enough, at multivariate analysis RAS blockers were significantly associated neither with renal survival, nor with TA-proteinuria. This is in disagreement with data obtained from randomised clinical trials in IgA nephropathy [25], probably reflecting a bias to treatment indication. Anyway the proportion of patients whom RAS blockers were prescribed was similar in the three different treatment groups. Then it seems to be as the fast decrease of proteinuria we observed in the first 6 months was almost exclusively produced by either CS or CS+A treatment. On the contrary, the further small proteinuria reduction observed in the subsequent part of follow-up in all treatment groups may have been favoured by the increasing use of RAS blockers. It has been shown that when CS are added to RAS blockers, the benefit on renal survival and proteinuria significantly increases [26–27]. Therefore, we cannot exclude that the prevailing effect of corticosteroids on proteinuria could have partially masked the added benefit of RAS blockers.

Finally, our study as a number of limitations. First, this is not a pre-specified analysis, and this may reduce statistical power. Moreover, the design of the three different trials is not homogenous in respect of treatment schedules and patient characteristics, somehow complicating data interpretation. In addition, pts were enrolled in different time periods: differences in everyday clinical practice may have influenced patient outcome.

## Conclusions

According to the combined analysis of these three trials, TA-proteinuria is confirmed as one of the best outcome indicators in IgAN: the lower is TA-proteinuria, the higher is renal survival. This prognostic role is maintained in pts with severe renal insufficiency. Other factors related to renal survival are plasma creatinine and 24h proteinuria at baseline, treatment with CS or CS+A and BP values measured during follow-up. A 6-month course of corticosteroids seems the most effective therapy for reducing TA-proteinuria till a state of remission.

## Appendix 1

### Study participants

#### Steering Committee

C. Sarcina, F. Ferrario, B. Visciano, V. Terraneo, S. Furiani, C. Pozzi (Ospedale Bassini, Cinisello Balsamo, Milano), C. Tinelli and A. De Silvestri (Policlinico San Matteo, Pavia).

#### Investigators and Nephrology Departments

M. Garozzo, Ospedale S. Marta e S. Venera, Acireale; B. Vogt, Inselspital, Universitätsspital Bern, (Switzerland); P. Scaini, Spedali Civili, Brescia; A. Pani, Ospedale G. Brotzu, Cagliari; D. Spanti, Ospedale Cannizzaro, Catania; R. Cerutti, Ospedale Uboldo, Cernusco sul Naviglio, Milan; M. D’Amico, Ospedale S. Anna, Como; P. Pecchini, Istituti Ospitalieri, Cremona; M. Villa, Ospedale di Desio; L. Cirami, Ospedale Careggi, Florence; A.M. Savastano, Ospedale Universitaria, Foggia; L. Del Vecchio, Ospedale A. Manzoni, Lecco; M. Farina, Ospedale Maggiore, Lodi; C. Baraldi, Ospedale C. Poma, Mantova; G.B. Fogazzi, Ospedale Maggiore IRCCS, Milan; T. De Gasperi, Ospedale di Montichiari, Brescia; G. Ticca, Ospedale S. Francesco, Nuoro; L. Calvisi, Ospedale A.Segni, Ozieri; L. Amico, Ospedale V. Cervello, Palermo; L. Allegri, Ospedale Universitario, Parma; L. Semeraro, Ospedale Fondazione Maugeri IRCCS, Pavia; P. Finocchiaro, Ospedale CNR-IBIM, Reggio Calabria; R. Rustichelli, Ospedale S. Maria Nuova, Reggio Emilia; L. Pedrini, Ospedale Bolognini, Seriate; V. De Cristofaro, Ospedale di Sondrio; D. Santoro, Ospedale S. Vincenzo, Taormina; M. Alpa, CMID, Turin; S. Feriozzi, Ospedale Belcolle, Viterbo.

## Supporting Information

S1 File All the population characteristics are available in the supporting data file. (XLS)
